# Surgical Site Infections in Colorectal Cancer Surgeries: A Systematic Review and Meta-Analysis of the Impact of Surgical Approach and Associated Risk Factors

**DOI:** 10.3390/life14070850

**Published:** 2024-07-05

**Authors:** Valentin Calu, Catalin Piriianu, Adrian Miron, Valentin Titus Grigorean

**Affiliations:** 1 Elias University Emergency Hospital, 011461 Bucharest, Romania; 2 Department of Surgery, Carol Davila University of Medicine and Pharmacy, 020021 Bucharest, Romania; 3 “Bagdasar-Arseni” Clinical Emergency Hospital, 12 Berceni Road, 041915 Bucharest, Romania

**Keywords:** colorectal surgery, surgical complications, risk assessment, postoperative infections, perioperative management, surgical outcomes, meta-analysis, systematic review

## Abstract

Background: Surgical site infections (SSIs) represent a noteworthy contributor to both morbidity and mortality in the context of patients who undergo colorectal surgery. Several risk factors have been identified; however, their relative significance remains uncertain. Methods: We conducted a meta-analysis of observational studies from their inception up until 2023 that investigated risk factors for SSIs in colorectal surgery. A random-effects model was used to pool the data and calculate the odds ratio (OR) and 95% confidence interval (CI) for each risk factor. Results: Our analysis included 26 studies with a total of 61,426 patients. The pooled results showed that male sex (OR = 1.45), body mass index (BMI) ≥ 25 kg/m^2^ (OR = 1.09), American Society of Anesthesiologists (ASA) score ≥ 3 (OR = 1.69), were all independent risk factors for SSIs in colorectal surgery. Conversely, laparoscopic surgery (OR = 0.70) was found to be a protective factor. Conclusions: The meta-analysis conducted revealed various risk factors, both modifiable and non-modifiable, associated with surgical site infections (SSIs) in colorectal surgery. These findings emphasize the significance of targeted interventions, including optimizing glycemic control, minimizing blood loss, and using laparoscopic techniques whenever feasible in order to decrease the occurrence of surgical site infections in this particular group of patients.

## 1. Introduction

Colorectal cancer (CRC) is a multifactorial disease. It ranks as the third most prevalent malignancy and is the second leading cause of cancer-related fatalities worldwide [1,2,3]. The primary factors contributing to this disorder are genetic abnormalities that disrupt the balance of the colon and rectum tissues, particularly impacting the activity of oncogenes, tumor suppressor genes, and DNA repair mechanisms [2,4]. The disease usually originates as benign polyps or adenomas, which undergo a series of molecular changes that result in the development of malignancy [5]. CRC is prevalent in Western countries and its prevalence is also on the rise in Eastern Europe and Japan. Various risk factors have been found, such as advanced age, bad dietary patterns, tobacco use, inflammatory bowel disease, and genetic predispositions [6,7,8].

The pathogenic mechanisms encompass chromosomal instability, microsatellite instability, and CpG island methylator phenotype, which disrupt crucial signaling pathways such as WNT, MAPK/PI3K, and TGF-β [2,4]. 

Prognosis can be significantly improved by finding and removing adenomatous polyps via early diagnosis using screening methods like colonoscopy, sigmoidoscopy, and stool testing [6,7]. The choice of therapeutic technique is contingent upon variables such as the tumor’s site and stage, and the unique attributes of the patient. Typically, it encompasses surgical procedures, chemotherapy, radiation therapy, and targeted medications [9,10].

Advancements in molecular genetics have resulted in the discovery of several biomarkers, including MSI, RAS, BRAF, and TP53. These innovative insights significantly enhance the advancement of customized treatment options and lead to improved outcomes for individuals with CRC [10,11]. 

Although recent research has achieved substantial advancements, the current 5-year survival rate in the United States remains at around 65%. This underscores the imperative for continuous research and improved screening protocols in order to substantially reduce death rates [6]. 

Surgical site infections (SSIs) are one of the most common complications of surgical procedures, including those for colorectal cancer. SSIs can have serious consequences for patients, including prolonged hospital stays, increased morbidity and mortality, and increased healthcare costs [12,13,14,15]. In colorectal cancer surgeries, the incidence of SSIs has been reported to be between 5% and 30%, depending on the study [16,17]. The risk factors associated with SSIs in colorectal cancer surgeries are multifactorial and include patient-related, procedure-related, and environmental factors [17].

To improve patient outcomes and reduce healthcare costs, there has been increasing interest in identifying risk factors associated with SSIs in colorectal cancer surgeries [17,18,19,20], as well as exploring the impact of different surgical approaches on SSI rates [21,22,23]. Previous studies have identified several risk factors for SSI in colorectal cancer surgeries, including patient age, obesity, diabetes, smoking, and immunosuppression [24,25,26]. Additionally, there has been interest in comparing the rates of SSI between laparoscopic and open surgeries. While some studies have found lower SSI rates with laparoscopic surgery [27,28,29], others have found no significant differences between the two approaches [30]. 

Tumor resection is the primary treatment for colorectal cancer, and it is performed in approximately 90% of patients. Surgical site infections (SSIs) are a frequently encountered postoperative complication of colorectal cancer surgery that can significantly affect the quality of surgery and patient well-being. One study found that meticulous wound management during surgery can significantly reduce the risk of incisional SSIs in elective colorectal cancer surgeries [31] and several risk factors for SSIs were identified after elective resection for rectal cancer, including preoperative radiotherapy and blood transfusions [32]. According to Murray et al. [33], the likelihood of surgical site infections (SSIs) is contingent upon the site of the ailment and the specific segment of colorectal resection for cancer. Additionally, Vo et al. [34] discovered that the incorporation of oral antibiotics into mechanical bowel preparation can decrease the occurrence of SSIs following resections for left colon and rectal cancer.

Other studies have investigated the impact of surgical techniques on the incidence of SSIs in colorectal cancer surgeries and Chen et al. [35] found that the use of a dual-ring wound protector can reduce the incidence of SSIs after elective surgery for colorectal cancer [36]. Another study reported that laparoscopic colorectal resection is associated with a lower incidence of SSIs compared to open surgery [37]. Cerdán et al. [38] investigated the impact of laparoscopic surgery on the incidence of SSIs and found that it may decrease morbidity and length of stay after elective colon cancer resection, especially in frail patients. 

Although surgical site infections (SSIs) are frequently observed in colorectal cancer surgery, there is a dearth of consensus among extant research regarding the risk factors linked to this complication, leading to inconsistent outcomes. Consequently, a dearth of efficacious perioperative approaches exists for the prevention of surgical site infections (SSIs) in patients undergoing colorectal cancer surgery. 

This study conducts a thorough examination and compilation of existing evidence on the factors that contribute to the higher occurrence of surgical site infections (SSIs) in colorectal cancer surgery. Furthermore, it analyzes the various surgical techniques employed in these surgeries. The study provides a comprehensive understanding of the elements that contribute to SSIs by identifying both modifiable and non-modifiable characteristics. The findings highlight the importance of targeted interventions, such as enhancing pre- and post-operative care, implementing strategies to prevent infections, and investigating the potential benefits of laparoscopic techniques in reducing the occurrence of surgical site infections. This research contributes to the field by offering evidence-based recommendations that can inform decision-making.

Overall, the systematic review highlights the complex and varied causes of surgical site infections (SSIs) in colorectal cancer surgeries. It emphasizes the need to consider and manage both patient-related and treatment-related risk factors in order to decrease the occurrence of SSIs and enhance surgical outcomes.

## 2. Methodology

### 2.1. Search Strategy

A systematic search was conducted by following Preferred Reporting Items for Systematic Reviews and Meta-Analyses (PRISMA) [39] guidelines, using the Medline-PubMed, Cochrane Library, and EMBASE databases as of March 2023 (cut-off date: 28 March 2023). The following Medical Subject Heading (MESH) terms in the PubMed database were used: “(colorectal neoplasms) AND ([surgical wound infection OR surgical site infection] OR [infectious OR wound OR skin] complications) AND (risk factors OR diabetes mellitus OR obesity OR body mass index OR aged OR hypertension OR neoplasm staging OR operative time OR hypothermia OR anti-infective agents OR hypoproteinemia OR hand hygiene OR laparotomy OR laparoscopy)”. 

### 2.2. Inclusion and Exclusion Criteria

The PICOS categories (i.e., population, intervention, comparator, outcomes, and study design) were used to define study inclusion criteria. 

The following inclusion criteria were used for the meta-analysis:

(1) Patients who have undergone surgical intervention for colorectal cancer;
(2) Evaluation of the relationship between any risk factors and SSIs;
(3) A standardized definition of the outcome measure for SSIs is reported by the devoted staff;
(4) Reported odds ratios (ORs) or relative risks (RRs) for SSIs, along with matching 95% confidence intervals (CIs);
(5) Patients included >18 years old;
(6) Case-controlled or cohort studies.

The exclusion criteria were:

(1) Animal or in vitro studies;
(2) Review articles, case reports, letters, or conference abstracts;
(3) Duplicate publications;
(4) Studies with incomplete data;
(5) Studies that were conducted on benign lesions and did not include cancer patients
(6) Studies published in languages other than English.

### 2.3. Data Extraction and Quality Assessment

Two independent reviewers screened and assessed the eligibility of the identified studies for inclusion in the meta-analysis. Disagreements were resolved by consensus or by a third reviewer. Data were extracted using a standardized form, including study characteristics (authors, year of publication, country, study design, sample size, risk factor investigated, age, type of SSI, and location of resection). The methodological quality of the included studies was assessed using the GRADE scale for all included studies (Supplementary Materials Table S1).

### 2.4. Data Synthesis and Analysis

For our analysis, we utilized RevMan version 5.3 (Cochrane, London, UK) to perform the statistical evaluations. We applied a random-effects model, specifically the DerSimonian–Laird method, to calculate the overall odds ratio (OR) and 95% confidence interval (CI) for each risk factor identified. We considered a *p*-value of less than 0.05 to indicate statistical significance.

To assess heterogeneity among the studies, we employed Cochran’s Q test and quantified heterogeneity using the I^2^ statistic. We interpreted the I^2^ values as follows: low heterogeneity (<50%), moderate heterogeneity (50–74%), and high heterogeneity (>75%). In instances where significant heterogeneity was detected, we conducted sensitivity analyses to explore the potential sources of heterogeneity and adjusted our model accordingly.

We also examined potential publication bias using funnel plots. To address any asymmetry in these plots, we applied the trim-and-fill method, which allowed us to correct for publication bias and ensure the robustness of our results. Section 4 of our manuscript provides visual representations of these analyses.

### 2.5. Risk of Bias Assessment

Two independent authors (C.P. and V.C.) assessed the risk of bias in the included studies using the Cochrane revised instrument for assessing risk of bias in randomized trials (RoB 2) [40] (Supplementary Materials Table S2). The evaluation centered on five domains: bias resulting from randomization, bias resulting from deviations from intended interventions, bias resulting from lacking outcome data, bias in outcome measurement, and bias in the selection of reported results. Low risk of bias trials were those with “low risk of bias” across all domains, whereas high risk of bias trials had “uncertain risk of bias” or “high risk of bias” in one or more domains. Any discrepancies were resolved by repeating the evaluation or consulting with a third reviewer (A.M.).

### 2.6. Common Techniques and Datasets

We included data from observational studies that examined different procedures for colorectal surgery, such as laparoscopic and open surgeries. The datasets encompassed studies conducted in various countries and healthcare settings, thus facilitating a complete examination of worldwide patterns. The analysis focused on key approaches such as the utilization of wound retractors, perioperative antibiotics regimes, and strategies for glycemic management. 

The studies conducted a comparison between the outcomes of standard open operations and minimally invasive laparoscopic procedures, emphasizing the developments in surgical methods and their influence on surgical site infection (SSI) rates.

### 2.7. Treatment Methods

The treatment approaches assessed in the research covered a range of surgical and perioperative treatments aimed at reducing the likelihood of surgical site infections (SSIs). The methods encompassed: 

Surgical Techniques: The study examined both laparoscopic and open surgical techniques, specifically investigating their effects on surgical site infection (SSI) rates. The minimally invasive aspect of laparoscopic surgery was contrasted with the conventional open surgery approach.

The effectiveness of wound retractors, namely double-ring wound protectors, in preventing surgical site infections (SSIs) was evaluated in the context of wound management. The study also assessed the effectiveness of precise intraoperative wound management techniques, including the utilization of sterile barriers and continuous irrigation devices.

The study analyzed the timing, kind, and duration of antibiotic prophylaxis. The studies examined the differences between using a single dose or multiple doses of medication, as well as the effects of adding oral antibiotics to mechanical bowel preparation.

The study examined methods for improving glycemic control in both diabetic and non-diabetic individuals. This encompassed the influence of preoperative HbA1c levels and intraoperative blood glucose control on surgical site infection (SSI) rates.

Nutritional Support: The study examined the impact of preoperative nutritional status and interventions, such as the use of nutritional supplements and preoperative fasting regimes.

An analysis was conducted to examine the association between techniques used to decrease blood loss during surgery and the usage of blood transfusions, and the risk of surgical site infections (SSIs). The study also took into account the influence of perioperative blood management procedures on patient outcomes.

The effectiveness of postoperative wound care procedures, such as the utilization of closed suction drains and the implementation of enhanced recovery after surgery (ERAS) protocols, was assessed in order to determine their impact on lowering surgical site infections (SSIs).

## 3. Results

### 3.1. Study Characteristics

Two investigators conducted a comprehensive search of the Medline-PubMed, Web of Science, and Scopus databases up to March 2023. A total of 3136 studies were initially retrieved, of which 1786 remained after removing duplicates. After screening the titles and abstracts, 932 studies were excluded and the remaining 854 articles underwent full-text review by the two investigators. Finally, 26 studies met the inclusion criteria and were included in this meta-analysis. These studies were selected based on their relevance to the association between colorectal cancer surgery and SSI risk factors. The patient characteristics in the included studies were extracted and are summarized in Table 1.

**Figure 1:**
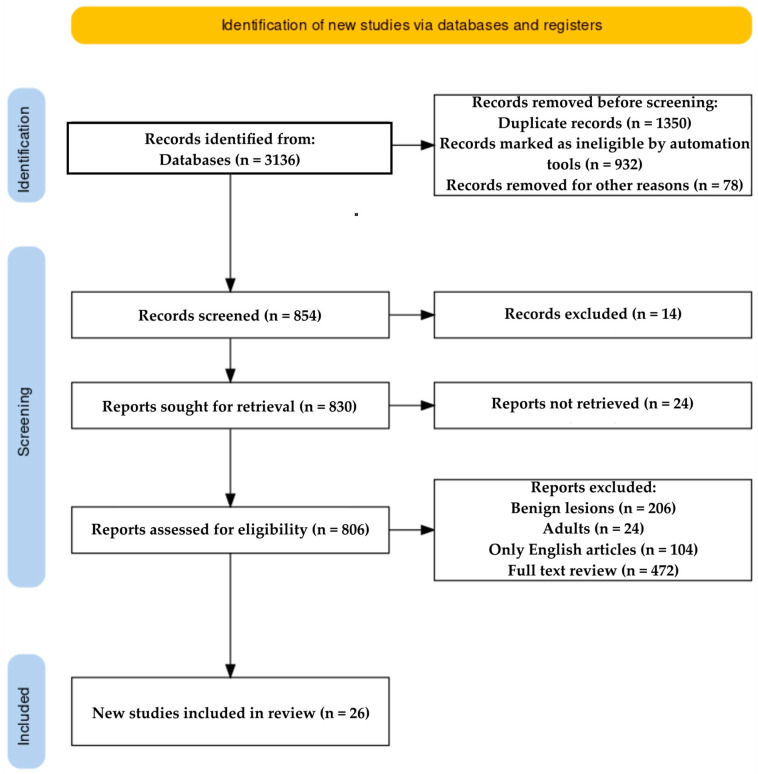
PRISMA flow Diagram.

The process of literature retrieval [57] is depicted in Figure 1.

### 3.2. SSI Risk Factors

Among the 26 papers that satisfied our inclusion criteria, we initially identified a total of 33 risk variables. Unfortunately, the absence of data prevented a quantitative analysis of 19 of these characteristics. For the remaining nine risk factors, which were mentioned in at least three articles, we performed a meta-analysis. Thereafter, these variables were divided into two groups: patient-related factors and treatment-related factors.

### 3.3. Patient-Related Risk Factors

#### 3.3.1. Diabetes Mellitus

**Figure 2:**
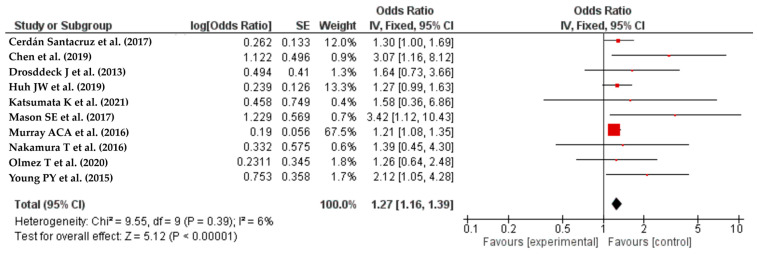
The forest plot shows the relationship between diabetes and colorectal resection SSIs. The red squares represent each study’s odds ratio (OR), with the size reflecting its meta-analysis weight. The 95% odds ratio CIs are horizontal lines next to the red squares. The diamond at the bottom of the plot shows the pooled odds ratio (OR) and 95% CI, which summarizes the combined effect of all studies. The study found a substantial correlation between diabetes mellitus and SSIs following colorectal resection, with an OR of 1.27 (95% CI: 1.16–1.39) and an I² value of 6% [33,35,38,41,43,44,45,47,49,55].

The analysis included ten studies [33,35,38,41,43,44,45,47,49,55] that demonstrated a significant positive association between diabetes mellitus and SSIs following colorectal resection, with an OR of 1.27 (95% CI: 1.16–1.39) and an I^2^ value of 6% (Figure 2).

Our investigation focused on assessing the influence of diabetes mellitus on the occurrence of surgical site infections (SSIs) after colorectal cancer operations. Diabetes mellitus is a widely recognized risk factor for postoperative complications, namely surgical site infections (SSIs). The articles included in our meta-analysis mainly pertain to diabetes mellitus without explicitly indicating the type (Type 1 or Type 2). Nevertheless, the focus of the evidence and clinical practice considerations primarily revolves around Type 2 diabetes mellitus (T2DM) because it is more commonly found in the general population and among patients who are undergoing colorectal procedures.

#### 3.3.2. Obesity

**Figure 3:**
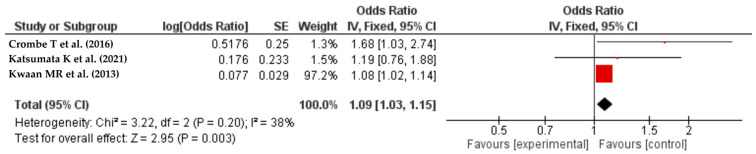
The forest plot shows how obesity affects colorectal resection surgical site infections (SSIs). The red squares represent each study’s odds ratio (OR), with the size reflecting its meta-analysis weight. The 95% odds ratio CIs are horizontal lines next to the red squares. The diamond at the bottom of the plot shows the pooled odds ratio (OR) and 95% CI, which summarizes the combined effect of all studies. Obesity was found to be a significant predictor of SSIs after colorectal resection, with an OR of 1.09 (95% CI: 1.03–1.15) and an I² value of 38% [42,45,46].

The study used the World Health Organization (WHO) classification to define obesity as having a BMI over 30 kg/m^2^. The studies [42,45,46] provided data on the association between obesity and SSIs in colorectal cancer surgeries. The meta-analysis revealed that patients with obesity may have a higher risk of SSIs, with an odds ratio (OR) of 1.09 and a 95% confidence interval (CI) of 1.03–1.15. The heterogeneity of the studies was evaluated using the ^I^ statistic, which showed moderate heterogeneity at 38%. The results are shown in Figure 3.

#### 3.3.3. Male Gender

**Figure 4:**
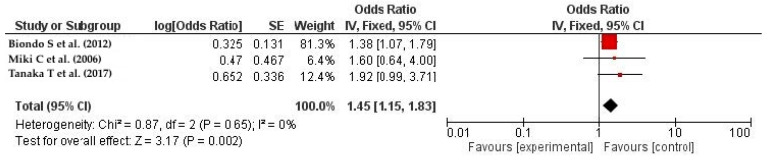
The forest plot shows that males are more likely to develop SSIs following colorectal resection. The red squares represent each study’s odds ratio (OR), with the size reflecting its meta-analysis weight. The 95% odds ratio CIs are horizontal lines next to the red squares. The diamond at the bottom of the plot shows the pooled odds ratio (OR) and 95% CI, which summarizes the combined effect of all studies. Males were significantly more likely to acquire surgical site infections (SSIs) following colorectal resection. The OR was 1.45 (95% CI: 1.15–1.83), therefore men were at higher risk. A 0% I² value indicates no significant heterogeneity among the analyzed studies. [32,48,50].

The analysis showed a significant association between male gender and SSIs, with an odds ratio of 1.45 and a 95% confidence interval of 1.15–1.83. The heterogeneity between studies was low (I^2^ = 0%), as shown in Figure 4 [32,48,50].

#### 3.3.4. ASA Score

**Figure 5:**
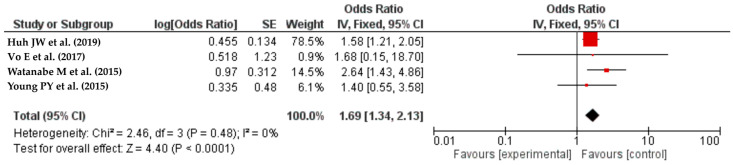
The forest plot shows shows that colorectal resection patients with an ASA score of 3 or higher are more likely to have SSIs. The red squares represent each study’s odds ratio (OR), with the size reflecting its meta-analysis weight. The 95% odds ratio CIs are horizontal lines next to the red squares. The diamond at the bottom of the plot shows the pooled odds ratio (OR) and 95% CI, which summarizes the combined effect of all studies. ASA scores of 3 or above were positively correlated with SSIs following colorectal resection [34,44,52,55].

A total of four studies [34,44,52,55] reporting on ASA classification were included in the meta-analysis. The results showed that patients with an ASA score of at least four had an increased risk of SSI (OR = 1.69, 95% CI: 1.34–2.13, I^2^ = 0%) (Figure 5).

### 3.4. Treatment-Related Factors

#### Laparoscopic Surgery

**Figure 6:**
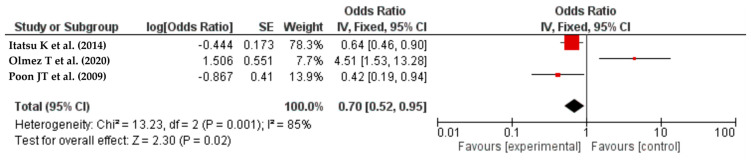
The forest plot shows how laparoscopic surgery increases the risk of SSIs after colorectal resection. The red squares represent each study’s odds ratio (OR), with the size reflecting its meta-analysis weight. The 95% odds ratio CIs are horizontal lines next to the red squares. The diamond at the bottom of the plot shows the pooled odds ratio (OR) and 95% CI, which summarizes the combined effect of all studies. Laparoscopy significantly reduces surgical site infections (SSIs). The OR was 0.70 (95% CI: 0.52–0.95), indicating a lower SSI risk. The I² score of 85% indicates significant heterogeneity among the analyzed studies [31,37,49].

A meta-analysis of three studies [31,37,49] indicated that patients who underwent selective laparoscopic colorectal cancer resection had a lower incidence of SSIs compared to those who underwent other surgical approaches (OR = 0.70, 95% CI: 0.52–0.95, I^2^ = 85%) (Figure 6). The prospective investigations conducted by Poon J.T. et al., and Itatsu K. et al., as well as the retrospective analysis conducted by Olmez T. et al., encompassed a cohort of patients who underwent elective colorectal surgery, encompassing both open and laparoscopic approaches. However, the patients were not randomly assigned to either the open surgery or laparoscopic surgery groups. The decision regarding the surgical strategy was made based on the patient’s request and the physician’s experience [31,37,49].

### 3.5. Stoma Creation

**Figure 7:**
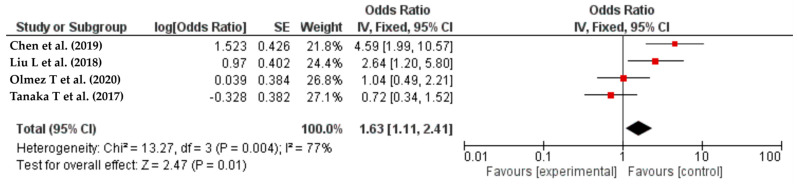
The forest plot shows the relationship between stoma creation and the risk of SSIs. The red squares represent each study’s odds ratio (OR), with the size reflecting its meta-analysis weight. The 95% odds ratio CIs are horizontal lines next to the red squares. The diamond at the bottom of the plot shows the pooled odds ratio (OR) and 95% CI, which summarizes the combined effect of all studies [35,49,50,54] The risk of surgical site infections (SSIs) increased by 1.63 (95% confidence interval: 1.11–2.41) after colorectal resection with a stoma. Evidence of 77% I² suggests moderate research.

A combined analysis of four studies [35,49,50,54] involving a total of 3221 participants showed that the risk of SSIs could increase by 203% with in-hospital stoma creation (OR = 1.63, 95% CI: 1.11–2.41, I2 = 77%) (Figure 7).

### 3.6. Wound Retractors

The use of wound retractors, particularly double-ring wound protectors, has shown a significant reduction in the incidence of surgical site infections (SSIs) following colorectal cancer surgeries. Multiple studies [58,59,60,61,62] have demonstrated the efficacy of these devices. An extensive examination of 18 randomized controlled studies involving 3744 patients revealed that the utilization of wound protectors after colorectal resection was associated with a reduced probability of surgical site infections (SSIs), with an odds ratio of 0.63 [60]. In addition, an additional study involving 2425 patients confirmed these findings, showing a similar reduction in the likelihood of surgical site infections (SSIs), with an odds ratio of 0.60 [63]. 

The Alexis wound retractor, a dual-ring shield, has proven to be highly effective, as research has indicated a significant decrease in surgical site infection (SSI) rates when compared to conventional methods. A study found that the Alexis group had no surgery site infections (SSIs), while the control group had an infection rate of 20% [58]. 

Furthermore, the CleanCision retractor, which integrates continuous antibiotic irrigation with barrier protection, demonstrated a much higher rate of success. The use of this technique resulted in a decrease in surgical site infection (SSI) rates to 1%, as opposed to the 9.4% rate observed with the Alexis retractor [62].

The implementation of circumferential wound retractors in emergency colorectal surgeries led to a significant reduction in surgical site infections (SSIs), as indicated by a *p*-value of 0.031 and an odds ratio of 8.5 [61]. On top of that, a novel wound retractor that integrates continuous irrigation and barrier protection was discovered to decrease total bacterial contamination by 66% and enteric bacterial contamination by 71%. Consequently, the incidence of surgical site infections (SSIs) was a mere 2.3% [59].

The cumulative results suggest that double-ring wound retractors, particularly those with additional features such as continuous irrigation, are highly effective in reducing surgical site infections (SSIs) in colorectal cancer surgeries. As a result, this results in improved patient outcomes and reduced healthcare costs.

#### Intraoperative Complications

**Figure 8:**
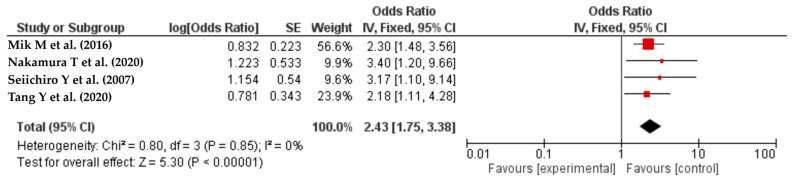
The forest plot shows the relationship between intraoperative complications and the risk of SSIs. The red squares represent each study’s odds ratio (OR), with the size reflecting its meta-analysis weight. The 95% odds ratio CIs are horizontal lines next to the red squares. The diamond at the bottom of the plot shows the pooled odds ratio (OR) and 95% CI, which summarizes the combined effect of all studies. The meta-analysis revealed a 152% increase in SSIs risk due to intraoperative complications (OR = 2.43, 95% CI: 1.75–3.38, I² = 0%) [26,51,56,64].

It is noteworthy that intraoperative complications were delineated as unforeseen unfavorable occurrences transpiring during the surgical procedure, encompassing iatrogenic harm to the bowel or blood vessels, hemorrhaging, intraoperative hypotension, malfunction of stapling devices, re-execution of anastomosis due to technical predicaments, intraoperative bacterial contamination, and other related incidents. The meta-analysis included four studies [26,51,56,64] that showed that the occurrence of intraoperative complications could increase the risk of SSIs by 152% (OR = 2.43, 95% CI: 1.75–3.38, I^2^ = 0%) (Figure 8).

## 4. Discussion

The results of the meta-analysis indicate that various factors are linked to a heightened likelihood of surgical site infections (SSIs). These factors include obesity, male gender, diabetes mellitus, an ASA score of ≥3, stoma creation, intraoperative complications, perioperative blood transfusion, and an operating time of ≥180 min. On the contrary, it was observed that laparoscopic resection of colorectal cancer exhibited a protective effect against surgical site infections (SSIs). The study also yielded a noteworthy finding that male patients exhibited a greater susceptibility to SSIs compared to their female counterparts, potentially attributable to gender-based variations in adipose tissue distribution. The presence of surplus visceral adipose tissue and abdominal adiposity in male patients may pose greater difficulty during surgical intervention and heighten the likelihood of surgical site infections [46,65].

Numerous studies have demonstrated that individuals who are overweight, obese, or morbidly obese are at a higher risk of developing surgical site infections (SSIs) compared to those with a normal weight. The increased risk is reported to be 1.2-fold, 1.5-fold, and 2.66-fold for overweight, obese, and morbidly obese individuals, respectively [36,53]. The data suggests a positive linear correlation between BMI and SSIs, despite the limitations of BMI as a measure of body fat composition. Additional variables, including subcutaneous fat thickness, visceral fat area, rectus abdominis thickness, and abdomen depth, may serve as more effective indicators for forecasting surgical site infections (SSIs) in individuals with colorectal cancer (CRC) [66].

Having a body mass index (BMI) below 18.5 kg/m^2^, which is considered underweight, has been associated with poorer outcomes in colorectal cancer (CRC) operations. This encompasses an increased probability of acquiring surgical site infections (SSIs). Studies indicate that underweight people have significantly lower overall survival rates compared to those who are not underweight, regardless of the stage of colorectal cancer (CRC). This highlights the vulnerability of underweight patients within this particular group [67,68]. More specifically, patients who are underweight and undergoing CRC surgery have demonstrated increased mortality rates. This can be related to their impaired nutritional state and decreased physiological reserves [68,69].

Malnutrition, which is commonly found in patients who are underweight, increases the likelihood of surgical site infections (SSIs) and other complications that occur after surgery. For example, hypoalbuminemia and hypoproteinemia, which are frequently observed in patients who are underweight, are important risk factors for surgical site infections (SSIs). This highlights the crucial role of maintaining a decent preoperative nutritional condition [70,71]. Furthermore, underweight individuals are more susceptible to prolonged hospital stays and a higher occurrence of medical and surgical complications, such as SSIs. This is due to their impaired immune systems and reduced wound-healing abilities [72]. While obesity is often acknowledged as a risk factor for surgery site infections (SSIs), being underweight also poses significant dangers, albeit through different mechanisms such as malnutrition and frailty [17,73,74]. 

To improve outcomes in underweight colorectal cancer (CRC) patients, it is crucial to implement effective nutritional therapy and do comprehensive preoperative assessments. These strategies are crucial for mitigating the risks linked with their inadequate nutritional condition [68,75]. Therefore, it is crucial to address the nutritional needs of underweight patients prior to undergoing CRC surgery in order to reduce the incidence of SSIs and improve overall surgical outcomes.

**Figure 9:**
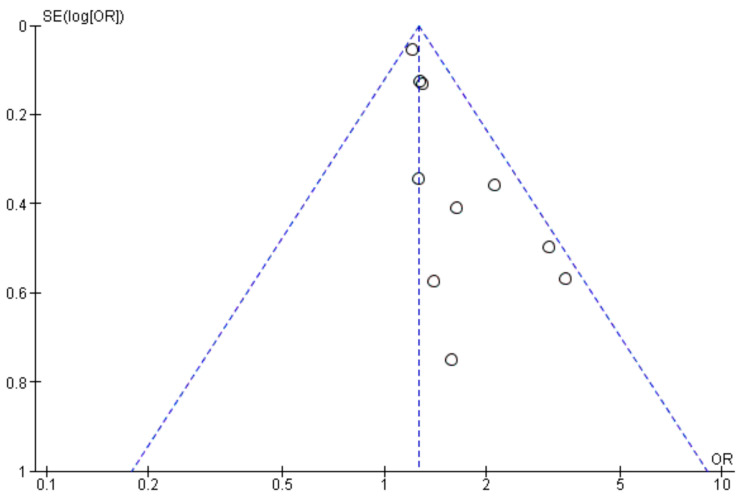
Funnel plot of Odds Ratios (OR) and Standard Errors (SE) for male gender and SSI risk. The x-axis represents the odds ratio (OR), and the y-axis represents the standard error (SE) of the log-transformed OR. Each dot represents an individual study. The dashed lines represent the 95% confidence limits around the summary effect estimate. The asymmetry in the plot suggests potential publication bias.

The meta-analysis (Figure 9) findings suggest that the male gender among colorectal cancer patients is associated with a 1.20 times higher likelihood of acquiring surgical site infections (SSIs) relative to the female gender. The observed variation could potentially be attributed to variations in fat distribution between genders, given that an overabundance of visceral fat and abdominal obesity in males may result in more intricate surgical interventions, extended surgical durations, and longer incisions, all of which may heighten the likelihood of surgical site infections [42,52,76,77].

Various patient characteristics have been examined as potential risk factors for surgical site infections (SSIs). However, due to the inconsistent findings reported in existing studies, the causal role of these factors is not widely acknowledged. In a prospective study conducted by Panos et al., a notable association was observed between individuals aged 70 and above and the occurrence of SSIs. [78]. The findings by Banaszkiewicz et al. [53] provided support for this claim; however, they were contradicted by the studies conducted by Kamboj et al., Hou et al., and Mu et al. [16,79,80]. The aforementioned studies have observed a significant inverse relationship between age and the risk of surgical site infections (SSIs). The authors of this research have postulated that this association may be attributed to surgeons exercising greater caution and refraining from employing invasive procedures when operating on older individuals. Similar to the aforementioned characteristics, a significant complicating factor arises from the diverse methodologies employed in the aforementioned investigations, wherein each study utilized distinct age thresholds ranging from 60 to 75 years.

Various comorbidities, such as obesity, diabetes mellitus (DM), cardiovascular disease (CVD), chronic obstructive pulmonary disease (COPD), and chronic kidney disease (CKD), have been found to be correlated with increased susceptibility to surgical site infections (SSIs) [27]. 

When considering surgical site infections (SSIs) that occur after colorectal cancer (CRC) procedures, it is important to differentiate between Type 1 diabetes mellitus (T1DM) and Type 2 diabetic mellitus (T2DM). Most of the literature and studies largely concentrate on T2DM because it is more common and has distinct consequences.

Type 2 diabetes mellitus (T2DM) significantly affects the incidence of surgical site infections (SSIs) in colorectal cancer (CRC) surgeries. Patients diagnosed with type 2 diabetes mellitus (T2DM) who have colorectal cancer (CRC) surgery are more prone to developing postoperative complications, such as surgical site infections (SSIs), compared to persons without diabetes. The increased susceptibility is attributed to characteristics such as raised blood glucose levels, excessive body mass, and reduced immune function, which are frequently observed in persons with diabetes [81,82]. 

Studies [83,84] have shown that preoperative glycated hemoglobin (HbA1C) levels, which indicate long-term glucose control, are independently associated with an increased risk of surgical site infections (SSIs) in colorectal cancer (CRC) treatments. Higher levels of HbA1C are directly correlated with an increased occurrence of SSIs. This underscores the significance of enhancing glycemic control prior to undergoing surgery. Moreover, individuals with diabetes often present a greater prevalence of comorbidities and reduced physical capacity, hence rendering their postoperative recuperation more arduous and elevating the likelihood of infection occurrence.

**Figure 10:**
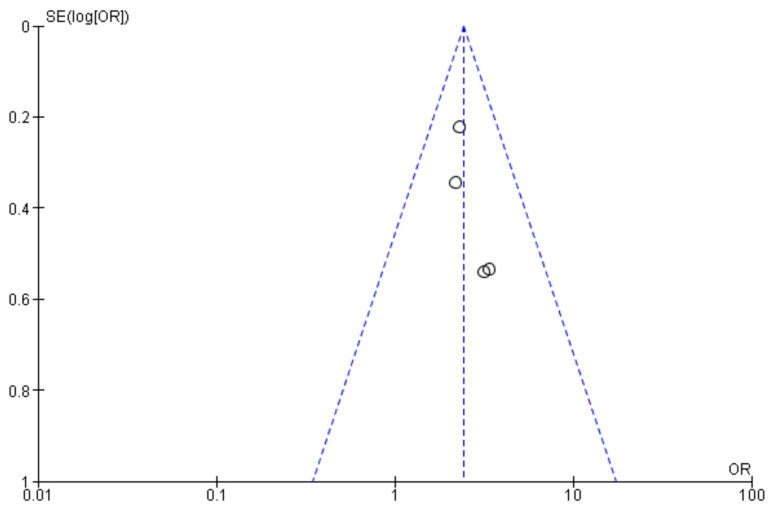
Funnel plot of Odds Ratios (OR) and Standard Errors (SE) for diabetes mellitus and SSI risk. The x-axis represents the odds ratio (OR), and the y-axis represents the standard error (SE) of the log-transformed OR. Each dot represents an individual study. The dashed lines represent the 95% confidence limits around the summary effect estimate. The slight asymmetry in the plot suggests potential mild publication bias.

Meta-analyses [85] (Figure 10) have confirmed that persons with diabetes have a much higher incidence of surgical site infections (SSIs), anastomotic leaks, and urinary issues following colorectal cancer (CRC) surgery. Moreover, the presence of T2DM has been linked to a higher rate of serious non-surgical postoperative complications and longer hospital stays, which can indirectly elevate the risk of SSIs [82,84]. 

Despite the lack of extensive studies specifically examining the impact of Type 1 diabetes mellitus (T1DM) on surgical site infections (SSIs) in colorectal cancer (CRC) procedures, we may deduce from a basic understanding of diabetes care and its associated consequences. Both Type 1 diabetes mellitus (T1DM) and Type 2 diabetes mellitus (T2DM) are characterized by elevated blood sugar levels and compromised immune function, which have a significant role in the occurrence of surgical site infections (SSIs). Therefore, it is crucial to effectively manage diabetes during the perioperative period to decrease the probability of surgical site infections (SSIs) and improve surgical outcomes for patients with colorectal cancer (CRC) [81,82,85]. 

Both immunosuppression and malnutrition are significant risk factors for surgical site infections (SSIs) that necessitate careful consideration. It is noteworthy that the impact of these disorders on the development of surgical site infections (SSIs) varies significantly depending on the specific surgical procedure. Chronic obstructive pulmonary disease (COPD) has been found to significantly elevate the likelihood of surgical site infections (SSI) subsequent to laparoscopic surgery. This can primarily be attributed to the disruption of pulmonary function parameters induced by the pneumoperitoneum. In contrast, it has been shown that immunosuppression and chronic kidney disease (CKD) have a propensity to promote surgical site infections (SSIs) in individuals undergoing open surgical procedures [27]. There is evidence to suggest that arterial hypertension and heart illness may also provide a predisposition to surgical site infections (SSIs) [86,87]. However, due to the presence of conflicting findings in the literature [43,88], it is imperative to conduct additional evaluations in order to achieve an appropriate and individualized approach to surgical therapy. 

As per the results of this investigation, the ASA score serves as a noteworthy parameter that mirrors the amalgamated comorbidities and physical states of individuals. Moreover, an ASA score of three or more is linked to an elevated susceptibility to SSIs [89]. Moreover, the investigation demonstrated that blood transfusion constitutes a self-sufficient hazard factor for surgical site infections (SSIs), given that allogenic transfusion-associated immunosuppression may constitute a plausible etiology for escalated SSI incidence [90]. Regrettably, there exist no alternative methods to avert the necessity of blood transfusions among surgical patients in order to mitigate the likelihood of surgical site infections (SSIs). Hence, it is imperative for surgeons to strive towards enhancing their surgical proficiency, lowering intraoperative hemorrhage, and diminishing the necessity for perioperative blood transfusions [91].

Furthermore, the risk of surgical site infection (SSI) is impacted by the underlying disease necessitating colorectal surgery. Specifically, it is seen to be higher in patients with inflammatory bowel disease or diverticulosis, but no significant association has been found in patients with neoplasms [92,93]. As per our investigation, a duration of 180 min or more in surgery was identified as an autonomous risk element for surgical site infection (SSI), aligning with several other scholarly works. Additionally, a noteworthy linear correlation exists between the duration of surgery and the likelihood of acquiring surgical site infections (SSIs). Specifically, there is a 13%, 17%, and 37% escalation in the occurrence of SSIs for every supplementary 15, 30, and 60 min of operative time, respectively [94,95]. The observed results may be attributed to prolonged exposure to environmental factors, local tissue injury, and the intricate nature of surgical procedures. Conversely, laparoscopic procedures for colorectal interventions have been determined to be both secure and efficacious, as evidenced by previous research [30]. In line with prior research, our combined data demonstrated a 34% decrease in the incidence of surgical site infections (SSIs) among individuals who received laparoscopic colorectal procedures in comparison to those who underwent laparotomy. This reduction in SSI is an autonomous protective element, as reported in previous studies [43,56]. 

It is imperative to consider and treat risk factors associated with both pre- and postoperative care. The factors encompassed in this category consist of the duration of hospitalization, administration of antibiotic prophylaxis, and the implementation of bowel preparation. A hospitalization duration of over 48 h prior to surgical intervention represents an indisputable risk factor for surgical site infections (SSIs), as it indicates a heightened severity of the patient’s medical condition and increases susceptibility to pathogenic microorganisms prevalent within the hospital setting [16,96]. The topic of preoperative bowel preparation remains a subject of ongoing discussion and analysis within the academic community. The majority of authors endorse a comprehensive strategy that involves the utilization of both mechanical bowel preparation and antibiotic prophylaxis. However, there is considerable variation in the selection of antibiotics and the method of administration [97]. In certain cases, it may be necessary to administer additional doses of antibiotics to high-risk patients both during and following surgical treatment.

Surgical procedures that are classified as urgent are linked to a heightened susceptibility to surgical site infections (SSIs) because of the patient’s deteriorated health status, lack of mechanical bowel preparation, and inadequate antibacterial prophylaxis [26]. 

Several studies have documented supplementary risk factors associated with surgical site infections (SSIs). These factors include persistent glucocorticoid use [32], chronic liver condition [31], congestive heart disease [62], respiratory illnesses [46,98,99], previous laparotomy [15,37,46,49,64,99,100,101,102,103,104], previous chemotherapy and radiotherapy [26,46,103], wound length and wound classification [31,46,103,105], intraoperative complications and surgeon’s abilities [106], and the lack of a wound protector [63]. Furthermore, the utilization of tobacco [47,102,107] has been recognized as a risk factor for surgical site infections (SSIs). Previous research has indicated that delayed wound healing may be linked to various factors such as prior laparotomy, chronic use of glucocorticoids, chronic liver disease, and cigarette smoking [45,47]. In addition, the dimensions of the injury, its categorization, and the nonexistence of a wound safeguard have the potential to lead to the infiltration of microorganisms into the abdominal wound. On top of that, empirical evidence suggests that the reduction in antibiotic concentration is attributed to hemorrhage [50,66]. Therefore, it is crucial for surgeons to avoid creating excessively large incisions and prioritize meticulous wound management in order to reduce the probability of surgical site infections (SSIs).

## 5. Conclusions

To summarize, the meta-analysis conducted identified several significant risk factors associated with surgical site infections (SSIs) in patients diagnosed with colorectal cancer (CRC). The variables that have been identified as significant factors in this study are body mass index (BMI), gender, American Society of Anesthesiologists (ASA) score, blood transfusion, duration of surgery, and surgical technique. The investigation revealed a number of plausible risk factors that may contribute to the incidence of surgical site infections. These factors include elevated blood sugar levels following surgery, tobacco use, prolonged use of glucocorticoids, chronic liver disease, congestive heart failure, respiratory ailments, prior laparotomy, previous exposure to chemotherapy and radiotherapy, wound length and classification, intraoperative complications, the surgeon’s level of experience, and the utilization of a wound protector.

The aforementioned results underscore the significance of personalized evaluation of preoperative risks, appropriate management of wounds, and efficient implementation of infection control strategies in order to mitigate the occurrence of surgical site infections in colorectal cancer surgery. Additional research is necessary to substantiate these risk factors and establish novel approaches for mitigating surgical site infections in patients with colorectal cancer.

## Figures and Tables

**Table 1:** Characteristics of included studies.

Study ID	Study Type	Country	Participants (n =)	Type of SSI	Techniques Used	Risk Factors Investigated
Itatsu K et al., 2014 [31]	Retrospective Cohort Study	Japan	1980	Incisional surgical site infections	Intraoperative Techniques, Care bundle	Intraoperative wound management
Nakamura T et al., 2020 [41]	Retrospective Cohort Study	Japan	1144	Wound infection	Preoperative and Intraoperative Techniques	Higher BMI, Longer operation time
Crombe T et al., 2016 [42]	Retrospective Cohort Study	France	1104	Postoperative infectious complications	Preoperative Mechanical Bowel Preparation, Intraoperative Care	Malignancy
Drosdeck J et al., 2013 [43]	Retrospective Cohort Study	USA	419	Surgical site infection	Laparoscopic, Antibiotics, Bowel Preparation	Multiple risk factors, including obesity, smoking, and wound classification
Huh JW et al., 2019 [44]	Retrospective Cohort Study	South Korea	3575	Surgical site infection	Laparoscopic, Open Surgery, Antibiotics, Bowel Preparation	High BMI, High ASA, Tumor location, Open surgery, Long operative time
Katsumata K et al., 2021 [45]	Retrospective Cohort Study	Japan	701	Surgical site infection	Lower rectal cancer, Mesorectal Excision, Lateral Lymph Node Dissection,	Male, Blood transfusions
Kwaan MR et al., 2013 [46]	Retrospective Cohort Study	USA	143	Superficial and deep incisional surgical site infection	Laparoscopic and Open Colectomy, Antibiotics	Abdominal wall thickness, smoking, alcohol use
Mason SE et al., 2017 [47]	Retrospective Cohort Study	UK	246	Surgical site infection	Peritoneal Insufflation with Warm, Humidified CO_2_	Postoperative hypothermia
Miki C et al., 2006 [48]	Retrospective Cohort Study	Japan	285	Site-specific surgical site infections	Wound Protectors	Male, Blood transfusions, Tumor location
Nakamura T et al., 2016 [41]	Retrospective Cohort Study	Japan	670	Surgical site infection	Laparoscopic, Antibiotics, Bowel Preparation	Diabetes mellitus, use of triclosan-coated PDS Plus sutures
Olmez T et al., 2020 [49]	Retrospective Cohort Study	Turkey	209	Surgical site infection	Laparoscopic, Open surgical approach, Antibiotics	Sarcopenia
Tanaka T et al., 2017 [50]	Retrospective Cohort Study	Japan	432	Surgical site infection	Bowel Preparation, Wound Protection	Preoperative nutritional status
Tang Y et al., 2020 [51]	Retrospective Cohort Study	China	326	Surgical site infection	Experienced surgeons, Laparoscopic	Chronic obstructive pulmonary disease, Abdominal surgical history
Watanabe M et al., 2015 [52]	Retrospective Cohort Study	Japan	538	Surgical site infection	Laparoscopic, Preoperative, and Intraoperative Measures	Visceral obesity, High BMI
Biondo S et al., 2012 [32]	Observational	Spain	2131	Surgical site	Surgical Techniques, Preoperative Measures	Male gender, Higher ASA, Tumor stage, Blood transfusion
Murray ACA et al., 2016 [33]	Retrospective cohort	United States	45,956	Surgical site	Surgical Approaches, Antibiotics, Intraoperative Measures	Disease location and colorectal resection segment
Banaszkiewicz Z et al., 2017 [53]	Retrospective cohort	Poland	1081	Surgical site	Surgical Approaches, Preoperative measures, Intraoperative Measures	Age, Comorbidities, Urgent surgery, Stoma
Vo E et al., 2017 [34]	Retrospective cohort	United States	191	Surgical site	Mechanical Bowel Preparation, Antibiotics, Preoperative skin preparation	The introduction of oral antibiotics into mechanical bowel preparation
Chen et al., 2019 [35]	Prospective randomized controlled trial	Taiwan	625	Surgical site	Dual-Ring Wound Protector	Use of dual-ring wound protector
Poon JT et al., 2009 [37]	Retrospective cohort	Hong Kong	1011	Surgical site	Bowel Preparation, Antibiotics, Skin Preparation, Wound closure	Blood transfusion
Liu L et al., 2018 [54]	Retrospective cohort	China	326	Not specified	Bowel Preparation, Antibiotics, Surgical Approaches, Wound protection	Preoperative anemia, Stoma
Ishikawa K et al., 2014 [36]	Retrospective cohort	Japan	224	Incisional	Preoperative, Intraoperative, and Postoperative Measures	Higher TNM, Intraoperative hypotension
Young PY et al., 2015 [55]	Retrospective cohort	South Korea	327	Surgical site	Antibiotics, Infection Control Measures, Preoperative skin preparation	Duration of prophylactic antibiotic use, Age, Nutritional status, smoking
Cerdán Santacruz et al., 2017 [38]	Observational	Spain	2968	Not specified	Laparoscopic and Open Surgery	Laparoscopic vs. open colon cancer resection
Seiichiro Y et al., 2007 [56]	Retrospective cohort	Japan	290	Wound	Bowel Preparation, Antibiotics, Laparoscopic	Stoma creation, Hypotension intraoperative, Operation length
Mik M et al., 2016 [26]	Prospective randomized controlled trial	Poland	2240	Surgical site	Bowel Preparation, Antibiotics, Surgical Approaches	Preoperative oral antibiotics, Obesity, Patient- and disease-dependent factors

## Data Availability

Data supporting the reported results are available in the following datasets: Medline-PubMed: https://pubmed.ncbi.nlm.nih.gov/ (accessed on 1 May 2023), Cochrane Library: https://www.cochranelibrary.com/ (accessed on 1 May 2023), EMBASE: https://www.embase.com/ (accessed on 1 May 2023).

## Supplementary Materials

The following supporting information can be downloaded at: https://www.mdpi.com/article/10.3390/life14070850/s1, Table S1: Quality of evidence presented in all included studies; Table S2: ROB assessment of all the included studies.
