# Supplementary material for: Surgical Site Infections in Colorectal Cancer Surgeries: A Systematic Review and Meta-Analysis of the Impact of Surgical Approach and Associated Risk Factors
Source: Life (Basel). 2024 Jul 5;14(7):850. doi: 10.3390/life14070850 (PMC11278392; doi:10.3390/life14070850)
Supplement: Supplementary file 1 [file life-14-00850-s001.zip › life-3026160-supplementary.docx]

**Table S1.** Quality of evidence presented in all included studies.

| **Study ID** | **Criteria** | | | | | | | | | | | | | **Overall appraisal** | |
| --- | --- | --- | --- | --- | --- | --- | --- | --- | --- | --- | --- | --- | --- | --- | --- |
| Itatsu K et al. 2014 (31) | ***C1*** | ***C2*** | ***C3*** | ***C4*** | ***C5*** | | ***C6*** | | ***C7*** | | *C8* | *C9* | |  | |
| Nakamura T et al. 2020 (64) | Y | Y | Y | Y | Y | | Y | | Y | | Y | N | | Included | |
| Crombe T et al. 2016 (42) | Y | Y | Y | Y | Y | | Y | | Y | | U | Y | | Included | |
| Drosdeck J et al. 2013 (43) | Y | Y | Y | Y | Y | | Y | | Y | | Y | Y | | Included | |
| Huh JW et al. 2019 (44) | Y | Y | Y | Y | Y | | N | | Y | | Y | Y | | Included | |
| Katsumata K et al. 2021 (45) | Y | Y | Y | Y | Y | | Y | | Y | | Y | U | | Included | |
| Kwaan MR et al. 2013 (46) | Y | Y | Y | Y | Y | | Y | | Y | | Y | Y | | Included | |
| Mason SE et al. 2017 (47) | Y | Y | Y | Y | Y | | Y | | Y | | Y | N | | Included | |
| Miki C et al. 2006 (48) | Y | Y | Y | Y | Y | | Y | | Y | | U | Y | | Included | |
| Nakamura T et al. 2016 (41) | Y | Y | Y | Y | Y | | Y | | Y | | Y | Y | | Included | |
| Olmez T et al. 2020 (49) | Y | Y | Y | Y | Y | | N | | Y | | Y | Y | | Included | |
| Tanaka T et al. 2017 (50) | Y | Y | Y | Y | Y | | Y | | Y | | Y | U | | Included | |
| Tang Y et al. 2020 (51) | Y | Y | Y | Y | Y | | Y | | Y | | U | Y | | Included | |
| Watanabe M et al. 2015 (52) | Y | Y | Y | Y | Y | | Y | | Y | | Y | Y | | Included | |
| Biondo S et al. (2012) (32) | Y | Y | Y | Y | Y | | N | | Y | | Y | Y | | Included | |
| Murray ACA et al. (2016) (33) | Y | Y | Y | Y | Y | | Y | | Y | | Y | U | | Included | |
| Banaszkiewicz Z et al. (2017) (53) | Y | Y | Y | Y | Y | | Y | | Y | | Y | Y | | Included | |
| Vo E et al. (2017) (34) | Y | Y | Y | Y | Y | | Y | | Y | | U | Y | | Included | |
| Chen et al. (2019) (35) | Y | Y | Y | Y | Y | | Y | | Y | | Y | N | | Included | |
| Poon JT et al. (2009) (37) | Y | Y | Y | Y | Y | | Y | | Y | | U | Y | | Included | |
| Liu L et al. (2018) (54) | Y | Y | Y | Y | Y | | Y | | Y | | Y | Y | | Included | |
| Ishikawa K et al. (2014) (36) | Y | Y | Y | Y | Y | | N | | Y | | Y | Y | | Included | |
| Young PY et al. (2015) (55) | Y | Y | Y | Y | Y | | Y | | Y | | Y | U | | Included | |
| Cerdán Santacruz et al. (2017)(38) | Y | Y | Y | Y | Y | | Y | | Y | | Y | Y | | Included | |
| Seiichiro Y et al. (2007) (56) | Y | Y | Y | Y | Y | | Y | | Y | | Y | N | | Included | |
| Mik M et al. (2016) (26) | Y | Y | Y | Y | Y | | Y | | Y | | Y | Y | | Included | |
| **Criteria** | | | | | | **Yes (Y)** | | **No (N)** | | **Unclear (U)** | | | **Not Applicable (NA)** | |  |
| 1. Was the study sample representative of the target population? | | | | | |  | |  | |  | | |  | |  |
| 1. Were study participants recruited appropriately? | | | | | |  | |  | |  | | |  | |  |
| 1. Was the sample size adequate? | | | | | |  | |  | |  | | |  | |  |
| 1. Were the study subjects and the study setting described in detail? | | | | | |  | |  | |  | | |  | |  |
| 1. Was the data analysis conducted with sufficient coverage of the identified population? | | | | | |  | |  | |  | | |  | |  |
| 1. Were objective, standard criteria used to measure the condition of interest? | | | | | |  | |  | |  | | |  | |  |
| 1. Was the condition measured reliably? | | | | | |  | |  | |  | | |  | |  |
| 1. Was there appropriate statistical analysis? | | | | | |  | |  | |  | | |  | |  |
| 1. Were all important confounding factors/subgroups/differences identified and accounted for? | | | | | |  | |  | |  | | |  | |  |
| 1. Were subpopulations identified using objective criteria? | | | | | |  | |  | |  | | |  | |  |

Yes (Y), No (N), Unclear (U),Not Applicable (NA).

**Table S2. ROB assessment of all the included studies.**

| **Itatsu K et al. 2014 (31)** |  |
| --- | --- |
| Risk of bias arising from the randomization process | Low |
| Bias due to missing outcome data | Low |
| Risk of bias in measurement of the outcome | High |
| Risk of bias due to deviations from the intended interventions | some concerns |
| Risk of bias in selection of the reported result | Low |
| Overall risk of bias | High |
| **Nakamura T et al. 2020 (64)** |  |
| Risk of bias arising from the randomization process | some concerns |
| Bias due to missing outcome data | Low |
| Risk of bias in measurement of the outcome | High |
| Risk of bias due to deviations from the intended interventions | Low |
| Risk of bias in selection of the reported result | High |
| Overall risk of bias | High |
| **Crombe T et al. 2016 (42)** |  |
| Risk of bias arising from the randomization process | Low |
| Bias due to missing outcome data | Low |
| Risk of bias in measurement of the outcome | High |
| Risk of bias due to deviations from the intended interventions | Low |
| Risk of bias in selection of the reported result | High |
| Overall risk of bias | High |
| **Drosdeck J et al. 2013 (43)** |  |
| Risk of bias arising from the randomization process | Low |
| Bias due to missing outcome data | Low |
| Risk of bias in measurement of the outcome | Low |
| Risk of bias due to deviations from the intended interventions | Low |
| Risk of bias in selection of the reported result | some concerns |
| Overall risk of bias | some concerns |
| **Huh JW et al. 2019 (44)** |  |
| Risk of bias arising from the randomization process | Low |
| Bias due to missing outcome data | Low |
| Risk of bias in measurement of the outcome | High |
| Risk of bias due to deviations from the intended interventions | Low |
| Risk of bias in selection of the reported result | Low |
| Overall risk of bias | High |
| **Katsumata K et al. 2021 (45)** |  |
| Risk of bias arising from the randomization process | Low |
| Bias due to missing outcome data | Low |
| Risk of bias in measurement of the outcome | Low |
| Risk of bias due to deviations from the intended interventions | Low |
| Risk of bias in selection of the reported result | Low |
| Overall risk of bias | Low |
| **Kwaan MR et al. 2013 (46)** |  |
| Risk of bias arising from the randomization process | Low |
| Bias due to missing outcome data | Low |
| Risk of bias in measurement of the outcome | Low |
| Risk of bias due to deviations from the intended interventions | Low |
| Risk of bias in selection of the reported result | Low |
| Overall risk of bias | Low |
| **Mason SE et al. 2017 (47)** |  |
| Risk of bias arising from the randomization process | Low |
| Bias due to missing outcome data | Low |
| Risk of bias in measurement of the outcome | Low |
| Risk of bias due to deviations from the intended interventions | Low |
| Risk of bias in selection of the reported result | Low |
| Overall risk of bias | Low |
| **Miki C et al. 2006 (48)** |  |
| Risk of bias arising from the randomization process | Low |
| Bias due to missing outcome data | some concerns |
| Risk of bias in measurement of the outcome | Low |
| Risk of bias due to deviations from the intended interventions | Low |
| Risk of bias in selection of the reported result | Low |
| Overall risk of bias | some concerns |
| **Nakamura T et al. 2016 (41)** |  |
| Risk of bias arising from the randomization process | Low |
| Bias due to missing outcome data | Low |
| Risk of bias in measurement of the outcome | Low |
| Risk of bias due to deviations from the intended interventions | High |
| Risk of bias in selection of the reported result | Low |
| Overall risk of bias | High |
| **Olmez T et al. 2020 (49)** |  |
| Risk of bias arising from the randomization process | Low |
| Bias due to missing outcome data | Low |
| Risk of bias in measurement of the outcome | Low |
| Risk of bias due to deviations from the intended interventions | High |
| Risk of bias in selection of the reported result | Low |
| Overall risk of bias | High |
| **Tanaka T et al. 2017 (50)** |  |
| Risk of bias arising from the randomization process | Low |
| Bias due to missing outcome data | Low |
| Risk of bias in measurement of the outcome | Low |
| Risk of bias due to deviations from the intended interventions | Low |
| Risk of bias in selection of the reported result | Low |
| Overall risk of bias | Low |
| **Tang Y et al. 2020 (51)** |  |
| Risk of bias arising from the randomization process | Low |
| Bias due to missing outcome data | Low |
| Risk of bias in measurement of the outcome | Low |
| Risk of bias due to deviations from the intended interventions | Low |
| Risk of bias in selection of the reported result | Low |
| Overall risk of bias | Low |
| **Watanabe M et al. 2015 (52)** |  |
| Risk of bias arising from the randomization process | Low |
| Bias due to missing outcome data | Low |
| Risk of bias in measurement of the outcome | Low |
| Risk of bias due to deviations from the intended interventions | Low |
| Risk of bias in selection of the reported result | Low |
| Overall risk of bias | Low |
| **Biondo S et al. (2012) (32)** |  |
| Risk of bias arising from the randomization process | some concerns |
| Bias due to missing outcome data | Low |
| Risk of bias in measurement of the outcome | Low |
| Risk of bias due to deviations from the intended interventions | Low |
| Risk of bias in selection of the reported result | Low |
| Overall risk of bias | some concerns |
| **Murray ACA et al. (2016) (33)** |  |
| Risk of bias arising from the randomization process | Low |
| Bias due to missing outcome data | Low |
| Risk of bias in measurement of the outcome | Low |
| Risk of bias due to deviations from the intended interventions | Low |
| Risk of bias in selection of the reported result | Low |
| Overall risk of bias | Low |
| **Banaszkiewicz Z et al. (2017) (53)** |  |
| Risk of bias arising from the randomization process | some concerns |
| Bias due to missing outcome data | Low |
| Risk of bias in measurement of the outcome | Low |
| Risk of bias due to deviations from the intended interventions | Low |
| Risk of bias in selection of the reported result | Low |
| Overall risk of bias | some concerns |
| **Vo E et al. (2017) (34)** |  |
| Risk of bias arising from the randomization process | Low |
| Bias due to missing outcome data | Low |
| Risk of bias in measurement of the outcome | Low |
| Risk of bias due to deviations from the intended interventions | Low |
| Risk of bias in selection of the reported result | Low |
| Overall risk of bias | Low |
| **Chen et al. (2019) (35)** |  |
| Risk of bias arising from the randomization process | Low |
| Bias due to missing outcome data | Low |
| Risk of bias in measurement of the outcome | Low |
| Risk of bias due to deviations from the intended interventions | Low |
| Risk of bias in selection of the reported result | Low |
| Overall risk of bias | Low |
| **Poon JT et al. (2009) (37)** |  |
| Risk of bias arising from the randomization process | some concerns |
| Bias due to missing outcome data | Low |
| Risk of bias in measurement of the outcome | Low |
| Risk of bias due to deviations from the intended interventions | Low |
| Risk of bias in selection of the reported result | Low |
| Overall risk of bias | some concerns |
| **Liu L et al. (2018) (54)** |  |
| Risk of bias arising from the randomization process | Low |
| Bias due to missing outcome data | Low |
| Risk of bias in measurement of the outcome | Low |
| Risk of bias due to deviations from the intended interventions | Low |
| Risk of bias in selection of the reported result | Low |
| Overall risk of bias | Low |
| **Ishikawa K et al. (2014) (36)** |  |
| Risk of bias arising from the randomization process | Low |
| Bias due to missing outcome data | Low |
| Risk of bias in measurement of the outcome | Low |
| Risk of bias due to deviations from the intended interventions | Low |
| Risk of bias in selection of the reported result | Low |
| Overall risk of bias | Low |
| **Young PY et al. (2015) (55)** |  |
| Risk of bias arising from the randomization process | Low |
| Bias due to missing outcome data | Low |
| Risk of bias in measurement of the outcome | Low |
| Risk of bias due to deviations from the intended interventions | some concerns |
| Risk of bias in selection of the reported result | Low |
| Overall risk of bias | some concerns |
| **Cerdán Santacruz et al. (2017) (38)** |  |
| Risk of bias arising from the randomization process | Low |
| Bias due to missing outcome data | Low |
| Risk of bias in measurement of the outcome | High |
| Risk of bias due to deviations from the intended interventions | Low |
| Risk of bias in selection of the reported result | Low |
| Overall risk of bias | High |
| **Seiichiro Y et al. (2007) (56)** |  |
| Risk of bias arising from the randomization process | Low |
| Bias due to missing outcome data | Low |
| Risk of bias in measurement of the outcome | Low |
| Risk of bias due to deviations from the intended interventions | Low |
| Risk of bias in selection of the reported result | Low |
| Overall risk of bias | Low |
| **Mik M et al. (2016) (26)** |  |
| Risk of bias arising from the randomization process | Low |
| Bias due to missing outcome data | High |
| Risk of bias in measurement of the outcome | Low |
| Risk of bias due to deviations from the intended interventions | Low |
| Risk of bias in selection of the reported result | Low |
| Overall risk of bias | High |
